# Does Inequality Blur Class Lines? Meritocratic Attitudes in Comparative Perspective

**DOI:** 10.1111/1468-4446.70107

**Published:** 2026-03-17

**Authors:** Roshan K. Pandian, Ronald Kwon

**Affiliations:** ^1^ Department of Sociology Southern Methodist University Dallas Texas USA; ^2^ Department of Sociology University of North Texas Denton Texas USA

**Keywords:** asymmetrical effects, class, cross‐national, income inequality, meritocratic beliefs, multilevel models

## Abstract

Scholars of inequality generally find that lower‐class individuals are more skeptical of meritocratic narratives that link economic success to individual work effort. However, past research has yielded inconclusive findings about how economic inequality affects meritocratic attitudes across different class groups. Theories of activated class conflict suggest that class matters more in high‐inequality contexts, while theories of relative power suggest that these class‐based differences are smaller in more unequal contexts. We argue that past studies have been unable to effectively adjudicate between these theories because they conflate the between‐ and within‐country relationships between inequality and meritocratic attitudes, assume that the impacts of rising and falling inequality are symmetrical, and tend to focus on rich democracies thus limiting important cross‐national and temporal variation in income inequality. Using multiple waves of the World Values Survey and multilevel regression models, we build on this prior research empirically by (1) disentangling the cross‐country and asymmetrical temporal components of the inequality‐meritocracy relationship, and (2) employing a broader sample of developing and rich countries to leverage more variation in country‐level income inequality. We find that class‐based cleavages in meritocratic attitudes are smaller in countries with higher average levels of inequality. Further, while *declining* inequality is associated with increased meritocratic beliefs across class lines, we find no evidence that *rising* inequality suppresses these beliefs. Our results demonstrate how contrasting theoretical frameworks can explain different components of the inequality‐meritocracy relationship (i.e., cross‐sectional and asymmetrical temporal components). We conclude by discussing the challenges our findings pose for partisans of egalitarian politics.

## 1 Introduction

Social scientists and activists have long been interested in how people make sense of inequalities around them (Kluegel and Smith 1986; Osberg and Smeeding 2006; McCall 2013; Newman et al. 2015; Solt et al. 2016; Mijs 2021). As income inequality has increased within rich democracies, many studies have counterintuitively found that residents of more unequal countries seem to express less concern about inequality^1^ (McCall 2013; Bucca 2016; Mijs 2021; Waight and Goldstein 2025). A likely explanation for this indifference is that individuals may increasingly see inequality as legitimate because it is perceived to arise from differences in how hard individuals work and reflect merit‐based outcomes—those with more income worked harder than those with less (Mijs 2021). Meritocratic beliefs are thus integral to the ideological foundation that justifies inequality and are a key determinant of people's concern for inequality and support for redistribution (Ahrens 2022; Pañeda‐Fernandez et al. 2026).

Consequently, a large empirical literature explores the determinants of meritocratic attitudes. In this body of work, scholars have paid particular attention to the role of social class and relative economic position in shaping individuals' beliefs about inequality and meritocracy (Hadler 2005; Curtis and Andersen 2015; McCoy et al. 2013; Morris et al. 2022). The bulk of recent empirical research lends support to interest‐based theories of meritocratic beliefs which suggest that low‐ and working‐class individuals are more likely to reject meritocratic narratives of success, because these individuals are more cognizant of barriers to mobility and are likely more disenchanted with the economic system compared to upper‐class individuals.

However, scholars disagree about how these class‐based differences vary by *contextual* income inequality. Theories of activated class conflict suggest that high‐inequality contexts trigger class consciousness in ways that push lower‐class individuals to reject meritocratic narratives, while upper class individuals embrace meritocratic ideas of hard work to justify their economic position (Newman et al. 2015). According to this perspective, class‐based cleavages in meritocratic attitudes are larger in high‐inequality contexts. Competing theories linked to relative power perspectives suggest that these class‐based differences shrink in high‐inequality contexts, as economic elites are better resourced and more successful in disseminating meritocratic narratives widely (Solt et al. 2016; Morris et al. 2022).

A number of recent empirical studies have attempted to adjudicate between these different theories about how income inequality and class are related to meritocratic attitudes, but as we discuss later, this research has generally yielded mixed findings (Newman et al. 2015; Solt et al. 2016; Morris et al. 2022; Mijs 2021; Bartram 2023). We argue that this valuable literature could be advanced by addressing some important limitations. First, most previous studies do not disentangle the between‐ and within‐country relationships between inequality and meritocracy (Newman et al. 2015; Solt et al. 2016; Morris et al. 2022). This is important because cross‐country patterns of inequality and meritocratic attitudes could have different implications than the temporal within‐country relationship between these variables. Further, the few studies that do disentangle the between‐ and within‐country components of the inequality‐meritocracy relationship assume that the temporal component is symmetric, meaning that rising and falling inequality have equal but opposite associations with meritocratic attitudes (Mijs 2021; Bartram 2023). For example, Bartram (2023) interprets a negative temporal relationship between income inequality and meritocratic attitudes as evidence that rising inequality suppresses meritocratic attitudes. However, as we discuss later, assuming that the impact of rising *and* falling inequality are equal but opposite risks conflating distinct processes that affect these attitudes differently. In this study, we decompose the inequality‐meritocracy link into its between‐ and within‐country (temporal) components but additionally, we rely on recent methodological advances to relax the symmetry assumption and further decompose the temporal relationship into its asymmetrical components (i.e., rising and falling inequality within countries). This strategy allows us to more effectively test different theories of how inequality and class are related to meritocratic attitudes. It is for example possible that past findings that support one theory (e.g., relative power theory) were driven by the between‐country component of these relationships, but the temporal components support alternative theories.

**FIGURE 1:**
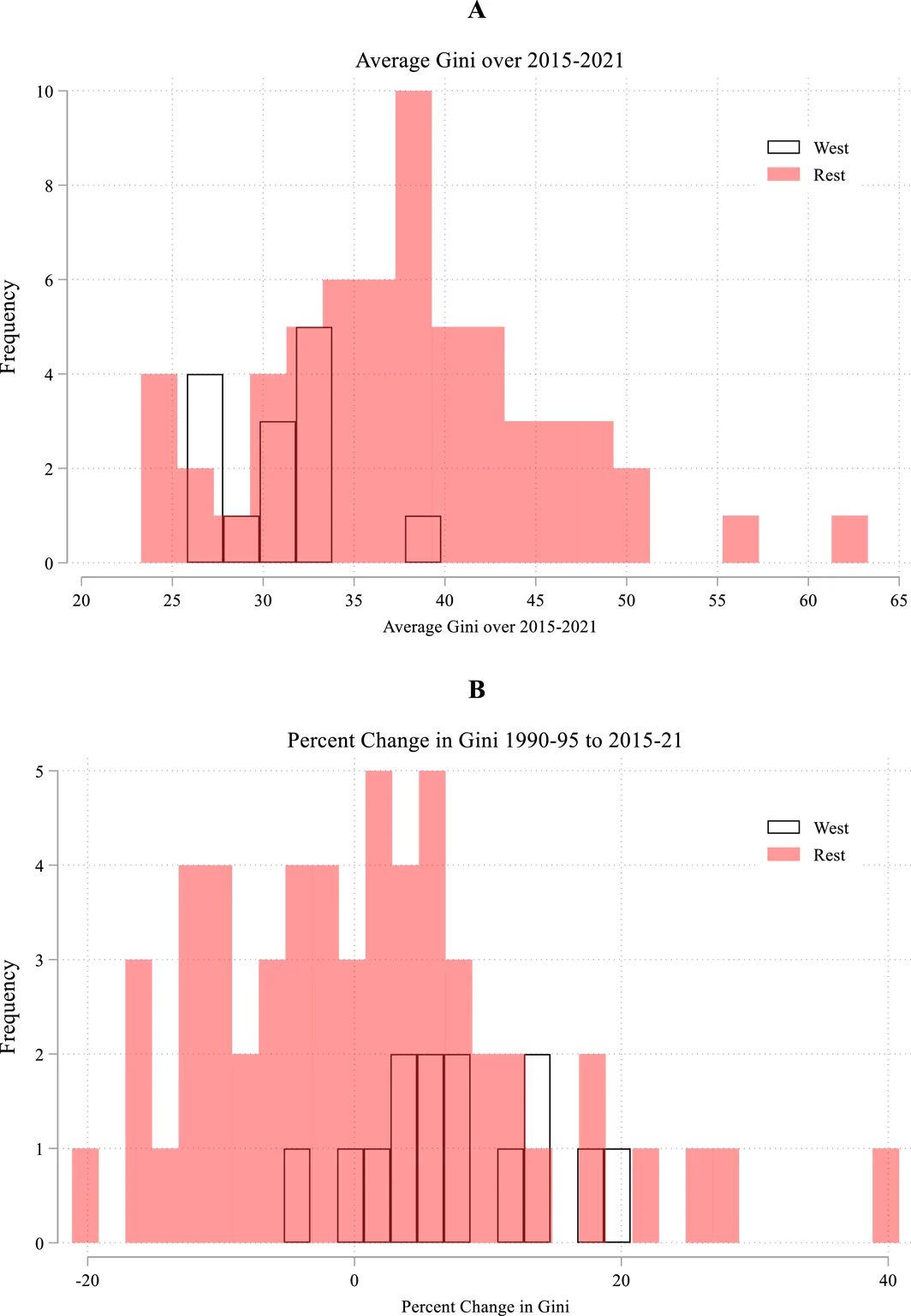
Levels and changes in national income inequality, west and the rest.

Second, virtually all cross‐national studies in this literature tend to focus exclusively on rich Western democracies (Newman et al. 2015; Solt et al. 2016; Mijs 2021; Morris et al. 2022; but cf. Bucca 2016). Bucca (2016) is an exception because it is a comparative study of Latin American countries. Still, given that the study examines data from only seven countries, it is not able to formally test relationships with country‐level predictors. This focus on rich nations is important for three main reasons. First, income inequality in non‐Western countries tends to be higher than in the West on average (Clark 2019, 2020; Pandian 2024). We illustrate this point in Panel A of Figure 1, which depicts the average Gini coefficient of countries analyzed in this study over the period 2015–2021. As this figure shows, non‐Western countries on average tend to have higher levels of inequality and exhibit more variation in inequality levels. Second, non‐Western countries also exhibit more variation in inequality *trends* over the past 3 decades as shown in Panel B of Figure 1. Third, alongside key differences in income inequality profiles, prior studies also demonstrate that aggregate meritocratic attitudes also tend to be lower in non‐Western countries compared to the West (Roex et al. 2019). As a result, existing studies omit key sources of variation that limit the generalizability of findings and undermine statistical power. In this study, we use a broad sample of countries rich and developing nations from the World Values Survey (WVS) that spans the past 3 decades to examine the question of how inequality and social class are related to meritocratic attitudes. In doing so, we more effectively leverage additional variation in a key predictor (income inequality) to answer this question.

Multilevel regression models of meritocratic attitudes from the WVS (1994–2022) uncover novel findings regarding class, inequality, and merit attitudes. We find that while lower class individuals are more skeptical of meritocratic narratives relative to upper class individuals, these class‐based differences in meritocratic attitudes are *smaller* in countries that are on average more unequal. In other words, consistent with relative power theories and several past studies, our results suggest that between‐country inequality (i.e., cross‐sectional component) is positively related to meritocratic attitudes, especially among lower‐class individuals. However, the *temporal* relationships we uncover between inequality and merit attitudes (i.e., within‐country components) are not consistent with relative power theories and are partly aligned with activated class conflict theories. We find that declining inequality is associated with increased meritocratic attitudes across class lines as individuals may come to view the economic system as more fair or legitimate. However, we do not find symmetrical evidence that rising inequality suppresses people's belief in meritocracy. We then conclude by discussing how our results speak to existing theories of meritocratic attitudes and how cross‐country patterns and temporal dynamics of inequality end up reinforcing merit attitudes in ways that pose an important challenge to egalitarian politics. In what follows, we provide an overview of past research in this area before describing the empirical analyses of this study.

## 2 Social Class and Meritocratic Attitudes

An extensive social science literature examines the relationship between individuals' social class position and their attitudes toward meritocracy and inequality (e.g., Hadler 2005; Curtis and Andersen 2015; Bucca 2016; Solt et al. 2016; Mijs 2021; Morris et al. 2022; Kwon and Pandian 2024; Zhu 2025). Interest‐based explanations suggest that working‐class individuals are especially disillusioned with an economic system that relegates them to the bottom rungs of society and this leads them to reject meritocratic explanations that locate the source of inequalities in individuals' talents and propensities to work hard (Downs 1957; Sears et al. 1980; Kluegel and Smith 1986; Lau 2003; Newman et al. 2015; Curtis and Andersen 2015). Theories of status and identity such as the “underdog principle” generate similar predictions: through shared experiences with economic hardship, lower‐class individuals are thought to develop sympathy for and solidarity with other low‐status individuals as they experience and become cognizant of rigid hierarchies and marginalization (Robinson and Bell 1978; Hadler 2005; Hunt 2007). Similarly, these theories also suggest that upper class individuals are likely to self‐servingly view the outcomes of the economic system as just and legitimate (Kluegel and Smith 1986; Osberg and Smeeding 2006). In other words, advantaged classes are more likely than others to hold meritocratic attitudes in order to legitimize the social processes that deliver them higher incomes and more wealth. Collectively, interest‐based and status theories arrive at similar hypotheses: that lower‐ and working‐class individuals are more likely to reject meritocratic explanations of inequality that emphasize hard work in favor of structural explanations or other non‐merit factors that focus on luck or an individual's family wealth and social connections.

In contrast, “system justification theory” (SJT) and neo‐Marxist theories of ideological hegemony and false consciousness predict that despite a seemingly clear incentive to reject meritocratic values and challenge the current economic system, lower class individuals are just as likely as upper class individuals to endorse meritocratic values (i.e., social class is likely unrelated to meritocratic attitudes) (Pines 1993; Jost and Banaji 1994; Jost and Orsolya 2005). Theories of false consciousness and ideological hegemony argue that “ruling class” ideas that justify economic inequalities are internalized by working class individuals because the dominant institutions of ideological production themselves reflect the power and dominance of economic elites (Pines 1993; Mijs 2016). Consequently, exploitation and oppression are obscured, and ruling class ideas become the ruling ideas of society. Building on ideological hegemony and neo‐Marxist theories, system justification theory (SJT) holds that individuals have a psychological need to view the world around them as predictable, desirable, and fair. Crucially, SJT suggests that individuals favor the current status quo system, and for working class individuals, belief in meritocracy provides comfort by casting the inequalities around them as legitimate, and implying that advancement is possible for everyone, even themselves (Jost et al. 2004; McCoy et al. 2013). For some scholars, the potential for upward mobility inherent in meritocratic views allows individuals to escape the subjective sense of economic powerlessness experienced by working class groups (Solt et al. 2016; Morris et al. 2022). In sum, these theories suggest that class position is unrelated to the adoption of meritocratic attitudes.

Past empirical research on meritocratic attitudes tends to find support for interest‐based explanations: lower‐class individuals hold less meritocratic views compared to upper class individuals (Curtis and Andersen 2015; Newman et al. 2015; Solt et al. 2016; Mijs 2021; Morris et al. 2022; but cf. Bucca 2016). According to these studies, individuals employed in working class occupations or earning lower incomes are less likely to hold meritocratic beliefs that legitimize inequality. However, this literature also suggests that the individual‐level relationship between class and meritocratic attitudes crucially depends on the level of *contextual* inequality.

## 3 Contextual Inequality and Class

Several past studies explore the relationship between contextual levels of inequality (e.g., national, regional, or local levels) and individuals' meritocratic attitudes (Newman et al. 2015; Solt et al. 2016; Mijs 2021; Morris et al. 2022). These studies advance competing theories about how this relationship varies by individual social class. Newman et al. (2015) posit an “activated class conflict” theory in which high inequality contexts “activate” latent opinions about merit and inequality that lead individuals to defend or attack the legitimacy of the economic system depending on whether they think it benefits or constrains their economic chances. In more unequal societies, relative deprivation and social comparison force lower‐class individuals to confront economic inequalities in their daily lives and to become more cognizant of their socioeconomic position (Festinger 1954; Runciman 1966; Alderson and Katz‐Gerro 2016; Walasek and Brown 2016). Ascribing their relatively lower status to factors outside of their own control (e.g., bad luck or that high status groups rely on social networks) rather than to personal shortcomings then becomes a way of protecting their self‐esteem (Cast and Burke 2002).

Similarly, upper‐class individuals are likely more class‐conscious in high‐inequality contexts than they otherwise would be in low‐inequality contexts. They are also likely to believe that the stakes are higher in a society where upper‐class groups control a larger share of income and as a result, are more likely to adopt meritocratic beliefs that legitimize their privileged position. In other words, higher contextual income inequality ratchets up class‐based cleavages in meritocratic attitudes because lower class individuals hold less meritocratic ideas about economic success/failure while upper‐class individuals are more likely to embrace these ideas. Relatedly, Waight and Goldstein (2025) find that while actual income inequality in the US grew substantially from the 1990s through the 2000s, the share of Americans perceiving that inequality was rising paradoxically declined, driven primarily by middle‐ and upper‐SES Republicans increasingly disavowing that inequality was growing. Although there are clear parallels with individual rational interest‐based explanations, activated class conflict theories extend such theories by arguing that high‐inequality contexts amplify class‐based differences in meritocratic attitudes.

Conversely, relative power theory suggests that high‐inequality contexts attenuate class‐based differences by raising the acceptance of meritocratic ideology among lower class individuals (Gaventa 1980; Goodin and Dryzek 1980; Solt et al. 2016; Morris et al. 2022). High levels of inequality reflect a political economy where the rich have comparatively more power relative to the poor, which in turn affords economic elites more resources to deploy in disseminating self‐serving meritocratic ideas that legitimize the economic system. Working‐class groups may have fewer resources to mobilize countervailing ideological projects that subvert dominant meritocratic values related to hard work. In such high‐inequality contexts, economic elites may exert greater ideological control and low/working‐class groups may be more likely to internalize meritocratic ideas as they are consistently exposed to them. Additionally, since economic elites likely have more to lose from the erosion of meritocratic norms in society, they might work harder to disseminate these ideas to the broader public. In these contexts, economic elites would ideally like to justify inequality because as past research documents, it is perceptions of “unfair” inequality that generates more support for redistribution among non‐elites (Ahrens 2022).

Other scholarship suggests that in more unequal societies, the social worlds that the rich and poor inhabit are more segregated (Mijs 2021). In these contexts, individuals from different class backgrounds are more likely to remain in their class habitus and go to different schools, occupy different types of jobs, marry people from the same economic background, socialize within the same class group, and reside in segregated neighborhoods (Musterd 2005; Reardon and Bischoff 2011; Massey and Tannen 2016; Owens 2016). The increased physical and social distance between rich and poor in high‐inequality contexts prevents individuals from fully grasping the extent and structural roots of economic inequality in society. Instead, all groups take for granted meritocratic cultural scripts about the centrality of hard work for economic outcomes. For example, Mijs and Adaner (2024) use an agent‐based simulation to show that under segregation conditions that prevail in highly unequal societies, people are more predisposed to meritocratic attitudes (i.e., they underestimate the role of inherited advantages and emphasize individual ability in shaping economic fortunes). Taken together, these theories suggest that class‐based differences in meritocratic attitudes are smaller in contexts with higher inequality.

To summarize, while past studies have generally found that lower class individuals are more skeptical of meritocratic narratives, this literature advances different hypotheses about how economic inequality interacts with class to shape meritocratic attitudes. According to activated class conflict theories, income inequality should be negatively associated with meritocratic attitudes among lower‐class individuals and positively related to these attitudes among upper‐class individuals, resulting in larger class divides in merit attitudes in high‐inequality contexts. In contrast, relative power theory argues that income inequality is positively related to merit attitudes especially among lower‐class individuals, which results in smaller class divides in merit attitudes in high‐inequality contexts.

## 4 Extending Past Research

Several recent empirical studies test these competing theories using survey data on meritocratic attitudes (Newman et al. 2015; Solt et al. 2016; Morris et al. 2022; Mijs 2021; Bartram 2023). Newman et al. (2015) use data from the US and show that lower class individuals are more likely to reject meritocracy particularly in counties with higher income inequality. In other words, Newman et al. (2015) find support for the interest‐based theories at the individual‐level and activated class conflict theories at the contextual‐level. Employing a methodological critique of Newman et al. (2015) and Solt et al. (2016) find the opposite at the contextual level: that the class‐based differences in meritocratic attitudes are largest in low‐inequality counties so that class matters less as income inequality rises. Importantly, Solt et al. (2016) arrives at these conclusions by using a larger sample of individuals and counties and illustrates the need to include more variation in units. Similarly, Morris et al. (2022) use data from the UK and uncover a positive relationship between local income inequality and meritocratic beliefs among the poorest UK residents, which again leads to smaller class‐based differences in more unequal places. Liu and Wang (2026) find that in high‐inequality countries, education serve to legitimize inequality while in low‐inequality countries, secondary and higher education has the opposite relationship. Mijs (2021) uses survey data from a sample of 23 rich Western countries and finds residents of more unequal nations tend to hold more meritocratic views while class‐based differences across the inequality spectrum are minimal. Further, while Mijs (2021) finds some limited evidence that temporal (within‐country) changes in inequality are positively related to meritocratic attitudes, Bartram (2023) uses different survey data from a similar set of Western countries and finds a negative relationship. In other words, past empirical research has produced mixed findings regarding how contextual inequality and social class are related to meritocratic attitudes.

This research also has two main limitations. First and most importantly, past studies conflate the between‐country and within‐country temporal components of the inequality‐meritocracy relationship and also treat the temporal relationship as symmetrical. However, it is important to note that heterogeneity across the various components of this relationship may have disparate theoretical implications. Second, past studies of meritocratic attitudes and inequality almost exclusively focus on rich democracies, effectively limiting valuable variation in key variables in the analyses. In what follows, we elaborate on these limitations and propose several ways that we extend this past work to address them.

### 4.1 Between‐Country Versus Within‐Country Variation in Contextual Inequality

Although prior studies outline the importance of contextual factors in shaping the meritocracy‐class link, the relationship may be more complicated than previously thought. Most of the empirical studies discussed above do not decompose the inequality‐meritocracy link into its within‐country and between‐country components (Newman et al. 2015; Solt et al. 2016; Morris et al. 2022; Liu and Wang 2026). For example, is a hypothetical positive relationship between contextual inequality and meritocratic attitudes due to (1) more unequal countries on average being inhabited by residents with more meritocratic attitudes, or (2) rising inequality within countries being associated with rising meritocratic attitudes among residents? These are two distinct claims that have different implications even though they might be equally insightful. Therefore, multilevel analyses of individual attitudes might potentially yield more fruitful insights when we can decompose contextual inequality's effect into its between‐country and within‐country (longitudinal) components (Fairbrother 2014; Schmidt‐Catran 2016; Mijs 2021). It is possible that such a decomposition yields distinct relationships for each component that do not uniformly support one theory (e.g., relative power vs. activated class conflict theories).

### 4.2 Asymmetry in the Longitudinal Relationship Between Inequality and Meritocracy

Does rising inequality affect meritocratic attitudes in the same way that declining inequality does? The longitudinal component of the inequality‐meritocracy relationship assumes that this relationship is symmetrical by default (i.e., that rising inequality has an equal but opposite coefficient as declining inequality). However, there is no a priori reason to think that this assumption holds. For example, it is possible that a negative association between temporal changes in inequality and meritocratic attitudes is driven by either (1) rising meritocratic attitudes when inequality declines, (2) declining meritocratic attitudes when inequality rises, or (3) both. In the first scenario, declining inequality might render the economic system more legitimate in the eyes of average individuals and strengthen elites' resolve to disseminate meritocratic ideas. In the second scenario, rising inequality undermines this legitimacy and suppresses meritocratic sentiments. It is quite possible that only one of the two scenarios described above is empirically true, which would mean that the relationship between inequality and meritocratic attitudes is asymmetric.

The two studies that decompose the between‐ and within‐country effects of inequality yield contradictory findings (Mijs 2021; Bartram 2023). Mijs (2021) provides some limited evidence that changes in inequality in Europe are positively related to meritocratic beliefs, but Bartram (2023) finds a negative relationship. Both studies interpret these positive and negative relationships in terms of the effects of *rising* inequality. However, as Bartram (2023) notes about his samples of countries, about half of the countries experienced a *decline* in inequality during the sample period. This leaves open the possibility that both studies are conflating the impact of rising and falling inequality by assuming a symmetrical temporal relationship between inequality and meritocracy. For example, is the negative relationship observed in Bartram (2023) being primarily driven by falling inequality spurring meritocratic attitudes or rising inequality suppressing them or both? In this study, we relax this symmetry assumption to help us more effectively adjudicate between theories of meritocratic beliefs. As we describe below, we use recent methodological guidance on modeling asymmetric temporal relationships in this study (Allison 2019).

### 4.3 Expanding the Sample of Countries

Studies of meritocratic attitudes have primarily focused on rich Western democracies and even the few studies that do include developing nations in their sample tend to analyze a single or small number of countries (e.g., Bucca 2016; Bubak 2019; Mijs and Hoy 2022; Kirsten and Biyase 2024). Further, virtually all cross‐national studies discussed above exclude low‐income countries from their analyses of the class‐inequality‐meritocracy relationship (Newman et al. 2015; Solt et al. 2016; Mijs 2021; Morris et al. 2022; but cf. Bucca 2016). This is important because, as depicted in Figure 1 in the introduction, developing nations tend to have higher levels of inequality and have experienced sharper changes in inequality over the past few decades. Crucially, Figure 1 also suggests that compared to rich countries, developing nations exhibit substantially more *variation* in inequality levels and trends over time. By omitting these countries, past cross‐national studies forego potentially useful additional variation in country‐level inequality that can be leveraged to test these theories. Additionally, such omission of large parts of the developing world limits the generalizability of the theories being tested.

In sum, past studies have produced conflicting findings regarding the role of social class and income inequality in shaping meritocratic attitudes. This study advances the empirical literature in several ways. First, we decompose the country‐level association between inequality and meritocratic attitudes into its between‐country and temporal within‐country components. Second, we further relax the assumption that the inequality‐meritocracy temporal relationship is symmetric, allowing us to distinguish between how meritocratic attitudes change when inequality rises versus when it falls. Third, we use survey data from an expanded sample of countries that includes non‐Western low‐income nations, and this affords us additional variation in contextual inequality for the analyses. In what follows, we describe the data and methods before presenting our results.

## 5 Data and Methods

### 5.1 Sample

The primary data source for this study is the World Values Survey (WVS), which provides nationally representative survey data for a broad range of countries (Inglehart et al. 2020). A key advantage of the WVS over other cross‐national surveys such as the International Social Survey Program (ISSP) is that the WVS covers a wider range of developing countries. In this study, we use waves 3 (1994–1998), 5 (2005–2009), 6 (2010–2014), and 7 (2017–2022) of the WVS. Waves 1, 2, and 4 were not used because key variables are missing for those waves. The main empirical analyses draw on a full sample of 264,859 individuals nested in 190 country‐waves and 85 countries. In robustness checks that use alternative measures of class (occupation and income‐based), the sample becomes considerably smaller due to these questions not being consistently fielded across survey waves.^2^ The appendix presents descriptive statistics (Table A1) for all the variables described below and a list of the countries included in the study (Table A2).

### 5.2 Outcome: Meritocratic Attitudes

Belief in meritocracy is measured using a WVS question that asks respondents to place their views on a 10‐point scale ranging from 1—“In the long run, hard work usually brings a better life” to 10—“Hard work doesn't generally bring success; it's more a matter of luck and connections.” We reverse‐code this variable so that higher values indicate stronger belief in meritocracy. This variable directly measures individuals' beliefs about how meritocratic they believe economic outcomes are based on the importance of “hard work.” This operationalization of meritocratic attitudes, based on beliefs about the relative importance of hard work, is consistent with recent studies that measure the same concept (e.g., Mijs 2021; Solt et al. 2016; Newman et al. 2015; Morris et al. 2022).

### 5.3 Focal Predictors

*Social Class*—We use a survey question that asks respondents to identify as either upper class, upper middle class, lower middle class, working class, or lower class. We dichotomize this variable coding as 1 to represent those respondents who identify as working class or lower class. This is a subjective perception‐based measure of social class. Rational interest‐based theories would expect that individuals who do not identify as lower‐ or working‐class should hold more meritocratic attitudes relative to those who do identify as such.

While individuals' self‐reports of their class position might be viewed as a less‐than‐ideal measure of class, recent cross‐national research suggests that subjective class identity is a substantially *better* predictor of household income and wealth compared to measures of class based on occupation and employment relations (Oesch and Vigna 2023). Past research also suggests that subjective class identification is particularly important for people's attitudes about inequality and support for redistribution (Franko and Witko 2023). Nevertheless, in robustness checks, we also test two alternative measures of class: an occupation‐based measure and another based on income. The WVS contains information about the characteristics of the respondent's occupation (e.g., business owner, manager, supervisor, professional, “unskilled, semi‐skilled, or skilled” worker, agricultural worker, farm owner, not employed etc.). We recode this variable into four categories: employer/manager, professionals, workers, and not in labor force. The “workers” category includes agricultural workers and other workers of all skill levels, both manual and non‐manual workers. Interest‐based theories would expect that workers are likely to hold less meritocratic ideas relative to employers/managers and professionals.

For the income‐based alternative measure of class, we rely on data from Donnelly and Pop‐Eleches (2018). The WVS asks respondents to place themselves on a 10‐point scale with each decile corresponding to country‐specific income brackets. However, Donnelly and Pop‐Eleches (2018) have critiqued the relative income scales on international surveys such as the WVS on the grounds that for a number of countries, these scales often have a skewed distribution across income deciles.^3^ This leads to bunching of respondents on one end of the scale which potentially affects any estimates of the effects of relative income on attitudes. To address this, Donnelly and Pop‐Eleches provide adjusted income percentile estimates for each WVS income decile within each country‐wave up to wave 5. We use these income percentile estimates as an income‐based measure of class in robustness checks.

*Country‐level Income Inequality*—Consistent with past cross‐national research on income inequality, we use the Gini coefficient to measure country‐level income inequality (e.g., Mijs 2021; Mahutga et al. 2017). These data come from the widely used Standardized World Income Inequality Database (SWIID) which provides cross‐nationally and temporally comparable estimates of yearly Gini coefficients for a broad range of countries (Solt et al. 2016). We use the “net income” version of the Gini variable (i.e., based on post‐tax and transfer incomes). To allow time for attitude formation and reduce the impact of yearly fluctuations, we lag this measure by averaging the Gini for the 3 years preceding the survey year (e.g., average Gini of 2016, 2017, and 2018 for survey year 2019).

As discussed above, we decompose the income inequality covariate into its between‐country and within‐country longitudinal components (Fairbrother 2014; Schmidt‐Catran 2016). Let $x_{jt}$ represent the Gini coefficient for country *j* in year *t*. The between‐country component is estimated using the country‐specific mean, $\bar{x_{j}}$, while the within‐country component is estimated using a covariate obtained from subtracting the country‐specific mean from each Gini observation: $x_{jt}^{within}=x_{jt}-\bar{x_{j}}$. However, this within‐country component assumes a symmetric relationship between the predictor and outcome.

To relax the assumption of symmetry in the longitudinal inequality‐meritocracy relationship, we follow Allison's (2019) approach in decomposing $x_{jt}^{within}$ into cumulative increases and decreases in inequality. First, $x_{jt}^{+}$ and $x_{jt}^{-}$ are defined as follows:

$$x_{jt}^{+}=x_{jt}-x_{jt-1}\;\text{if}\;\left(x_{jt}-x_{jt-1}\right)>0,\text{otherwise}\;0$$

$$x_{jt}^{-}=x_{jt}-x_{jt-1}\;\text{if}\;\left(x_{jt}-x_{jt-1}\right)<0,\text{otherwise}\;0$$

where $x_{jt}^{+}$ represents the increase in the Gini coefficient in year *t* relative to the previous year for country *j* and $x_{jt}^{-}$ represents the decrease in the Gini coefficient between consecutive year for country *j*.^4^ Then, $z_{jt}^{+}$ and $z_{jt}^{-}$ are defined as follows:

$$z_{jt}^{+}=\sum_{s=1}^{t}x_{js}^{+}$$

$$z_{jt}^{-}=\sum_{s=1}^{t}x_{js}^{-}$$

where $z_{jt}^{+}$ represents the accumulation of all yearly increases in the Gini since *t = 1* and $z_{jt}^{-}$ represents the accumulation of all previous yearly decreases. Finally, the $z_{jt}^{+/-}$ variables are then mean‐deviated by country by subtracting their country‐specific means from these variables before entering the regression equation.^5^

In total, the relationship between income inequality and meritocratic attitudes is estimated using three covariates: (1) the country‐specific means representing the between‐country component ($\bar{x_{j}}$); (2) the mean‐deviated accumulation of increases in the Gini coefficient (${z_{jt}^{+}}_{within}$) which gives us the within‐country effect of rising inequality; and (3) the mean‐deviated accumulation of decreases in the Gini coefficient (${z_{jt}^{-}}_{within}$) which gives us the within‐country effect of falling inequality.

### 5.4 Controls

We use several control variables in the main analyses. At the individual level, we control for age, gender, employment status, marital status, education (attendance at primary, secondary, and tertiary levels), parental status, and whether the respondent identifies as religious. At the country‐year level, we control for the between‐ and within‐country components of GDP per capita (World Bank 2024). Additionally, we include survey year and regional dummy variables corresponding to major world regions (West, East Asia and Pacific, Eastern Europe and Central Asia, Latin America and Caribbean, Middle East and North Africa, South Asia, and Sub‐Saharan Africa).^6^ In robustness checks, we test additional country‐level variables such as population, the KOF globalization index, memberships in international non‐governmental organizations, democracy score, and religious majority dummies but the inclusion of these predictors did not alter the findings of this study.

### 5.5 Estimation Strategy

Given the nested structure of the dataset, we estimate three‐level linear mixed effects regression models of meritocratic attitudes in which individuals are nested in country‐years, which are in turn nested in countries. To account for within‐unit clustering, we include random intercepts at the country‐year and country levels. The inclusion of random effects at both country‐ and country‐year levels is important because failure to account for clustering at all levels leads to downward bias in the standard errors (Schmidt‐Catran and Malcolm 2016). The numbers of higher‐level units (i.e., 190 country‐years and 85 countries) in our sample are higher than the minimum number (30) required in multilevel models to arrive at reliable variance estimates for higher‐level coefficients (Bryan and Jenkins 2016).

Following recent methodological work on multilevel models that finds that the failure to include random slopes for lower‐order predictors involved in cross‐level interactions leads to anti‐conservative standard errors (Heisig and Schaeffer 2019), we include random slopes for class at the country‐year and country levels since our main hypotheses involve a cross‐level interaction between an individual‐level predictor (i.e., class) and a higher‐level predictor (i.e., country income inequality). As is conventional in multilevel analyses of individual attitudes, we lag the longitudinal components of country‐level predictors (Gini, GDP per capita) to allow time for attitude formation. To do so, we take the average value of the previous 3 years of the predictor relative to the survey year.^7^

Finally, as is standard in the cross‐national literature employing multilevel models of attitudinal outcomes, we caution against causal interpretations of the relationships tested in this study. We believe that the estimated between‐country relationship between the Gini and meritocratic attitudes provides important descriptive insights about cross‐national patterns in meritocratic attitudes. However, causal interpretations of this between‐country relationship could be plagued by endogeneity. The estimated within‐country relationships are purged of time‐invariant country‐specific unobserved heterogeneity and therefore provide a firmer ground for causal claims, but time‐varying unobserved factors could still threaten causal identification.

## 6 Results

Table 1 displays results from the main multilevel regression models of belief in meritocracy. Model 1 includes just the control variables: individual‐level characteristics, region and year fixed effects, and the between‐ and within‐country components of GDP per capita. Model 2 adds the country‐level inequality predictors of interest. There is some evidence of a positive relationship between the outcome and the between‐country inequality component (*p* < 0.10) which means that countries with higher *levels* of inequality across the period studied tended to have residents with more meritocratic views. We also find evidence for an asymmetrical relationship between *changes* in inequality and meritocratic attitudes. According to Model 2, a one‐point decrease in the Gini coefficient is associated with a 0.13 unit increase in the meritocracy scale (*p* < 0.001). However, we find no evidence that increases in inequality are associated with declines in meritocratic attitudes (*p* > 0.10). Taken together, results from Model 2 are consistent with declines in inequality legitimating the economic system in the eyes of individuals and/or strengthening elites' propensity to disseminate meritocratic ideas as a response. However, rising inequality does not seem to be associated with analogous countervailing processes. These results underscore the importance of considering the asymmetric nature of the inequality‐meritocracy longitudinal relationship.

**TABLE 1:** Coefficients from mixed effects regression of meritocratic attitudes on predictors.

	(1)	(2)	(3)
Country‐level predictors			
GDP per capita (ln) (between‐country)	−0.369***	−0.350***	−0.319**
	(0.105)	(0.102)	(0.102)
GDP per capita (ln) (within‐country)	−0.456+	−0.703**	−0.720**
	(0.243)	(0.256)	(0.259)
Gini average (between‐country)		0.020^+^	0.018
		(0.012)	(0.012)
Gini decrease (within‐country)		0.128***	0.142***
		(0.038)	(0.039)
Gini increase (within‐country)		0.063	0.093^+^
		(0.050)	(0.050)
Subjective class and cross‐level interactions			
Low‐ or working‐class identification (=1)^a^			−0.192***
			(0.027)
Low/Working class × Gini average (between)			0.011***
			(0.003)
Low/Working class × Gini decrease (within)			0.002
			(0.016)
Low/Working class × Gini increase (within)			−0.038*
			(0.018)

Full set of controls (see note)	Y	Y	Y
Var (country)	0.185***	0.176***	0.168***
	(0.051)	(0.048)	(0.049)
Var (country‐wave)	0.206***	0.188***	0.189***
	(0.030)	(0.028)	(0.029)
Var (individual)	7.49***	7.49***	7.43***
	(0.021)	(0.021)	(0.021)
Observations	264,859	264,859	249,251
Country‐waves	190	190	184
Countries	85	85	83

*Note:* Standard errors in parentheses. Models 2 and 3 include individual controls for age, gender, education, religious person, employment status, and parental status in addition to region and year fixed effects. All models include random intercepts for country and country‐years as well as random slopes for individual class predictors involved in cross‐level interactions.

^a^ Main effect of class computed with other predictors held at sample means.

^+^ *p* < 0.10.

^*^ *p* < 0.05.

^**^ *p* < 0.01.

^***^ *p* < 0.001 (two‐tailed test).

Model 3 adds the individual‐level subjective class variable along with the cross‐level interactions with inequality. According to this model, individuals in the average country who identify as lower or working class are more skeptical of meritocracy by about 0.19 units on the meritocracy scale compared to those who do not (*p* < 0.001). This is largely consistent with prior studies of rich countries which find that lower class individuals are less likely to hold meritocratic attitudes (Newman et al. 2015; Solt et al. 2016; Mijs 2021; Morris et al. 2022). The statistically significant positive interaction of lower class status with the between‐country component of inequality suggests that this class‐based difference in meritocratic attitudes becomes *smaller* in more unequal countries.

**FIGURE 2:**
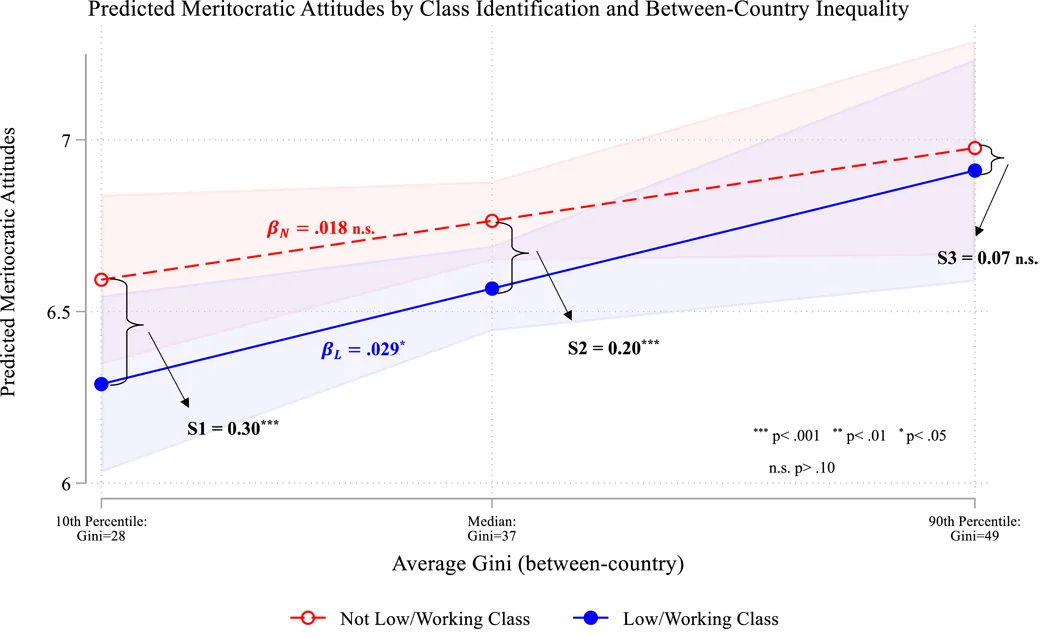
Predicted meritocratic attitudes by class identification and between‐country.

These patterns are illustrated in Figure 2 which depicts the predicted level of meritocratic beliefs by subjective social class and country‐level income inequality based on Model 3. In more egalitarian countries (i.e., Gini = 10^th^ percentile of sample ≈ 28; e.g. Netherlands or Kazakhstan in recent years), individuals who identify as low‐ or working‐class have lower levels of meritocratic beliefs compared to those who do not (*S*1 = 0.30, *p* < 0.001). This subjective class‐based difference shrinks to being statistically indistinguishable from zero (*S*3 = 0.07, *p* > 0.10) among those who reside in more unequal countries (i.e., Gini = 90^th^ percentile of sample ≈ 49; e.g. Brazil or Indonesia in recent years). Furthermore, this attenuation in class‐based differences is statistically significant at the 0.001 level (S1‐S3 = 0.24). Moreover, Figure 2 also shows that for individuals who do not identify as lower class, there is no statistically discernible relationship between country‐level inequality (between‐country) and meritocratic attitudes, while the same relationship for lower class individuals is positive and statistically significant ($\beta_{L}$ = 0.029, *p* < 0.05). These findings are consistent with relative power theories which suggest that high‐inequality regimes are more effective in widely disseminating meritocratic views, especially among lower class individuals.

**FIGURE 3:**
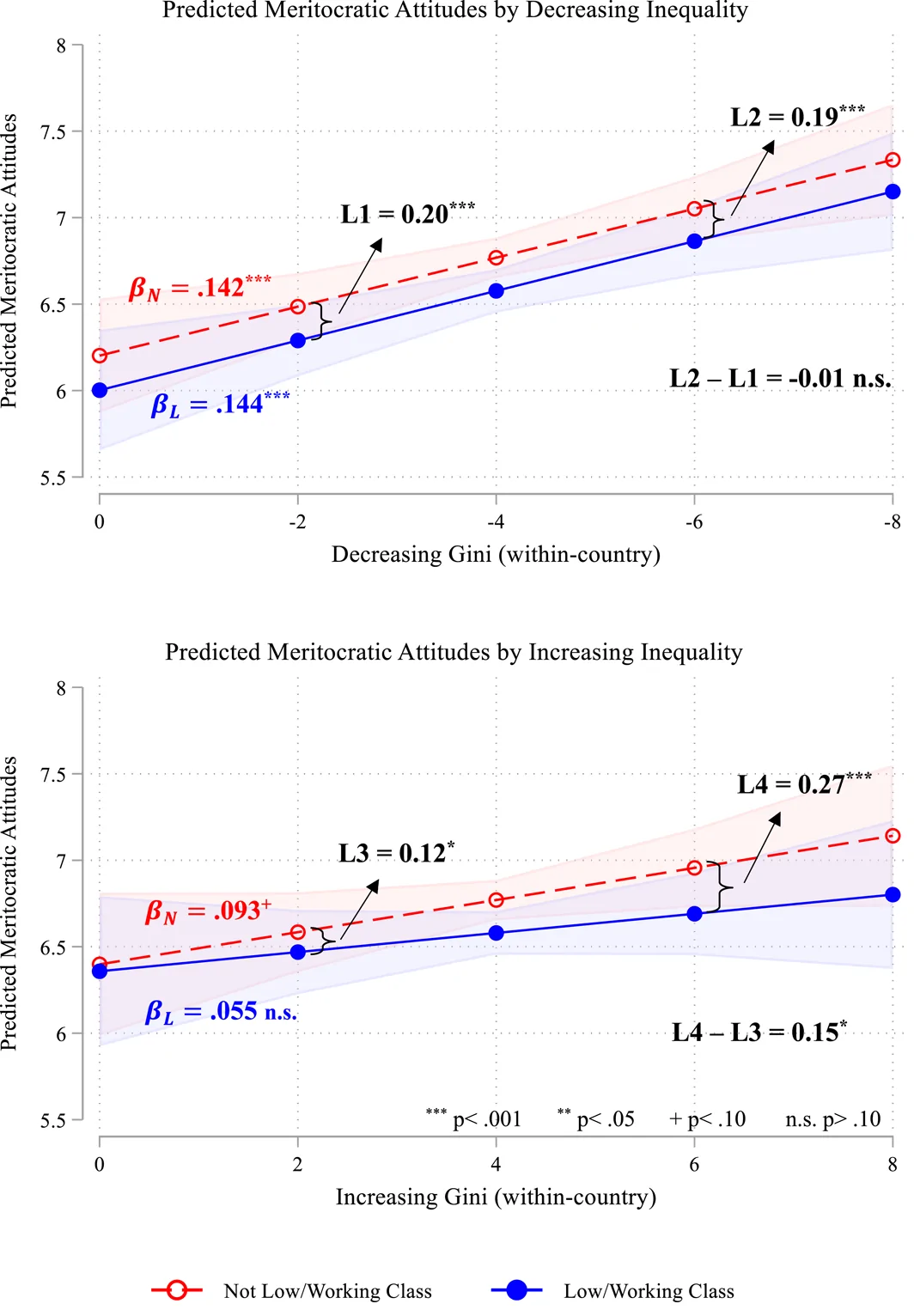
Predicted meritocratic attitudes by declining and rising inequality within countries.

Model 3 also suggests that declining inequality (within‐country) is associated with rising meritocratic attitudes, but this relationship does not vary by class. The small and non‐significant interaction term also suggests that class‐based cleavages do not vary by how much inequality declines. The top panel of Figure 3 plots the predicted level of meritocratic attitudes by the magnitude of inequality decline based on Model 3. A 1‐point decline in the Gini coefficient is associated with a 0.14‐point increase in the meritocracy scale (*p* < 0.001), and this relationship is virtually the same for both class categories. In contrast, the interaction between class identification and inequality‐*increase* suggests that class‐based cleavages become *larger* with rising inequality. This pattern is depicted in the bottom panel of Figure 3. The relationship between rising inequality and meritocratic attitudes is statistically indistinguishable from zero for lower class individuals (*p* > 0.10) but this relationship is larger (i.e., more positive) for those who do not identify as lower class ($\beta_{N}$ = 0.093, *p* < 0.10; $\beta_{N}-\beta_{L}$ = 0.038, *p* < 0.05). This finding provides suggestive evidence for activated class conflict theories, in which upper‐class individuals become more class conscious and likely to adopt meritocratic attitudes as inequality rises. However, lower class individuals seem resistant to adopting more meritocratic attitudes as inequality rises, leading to larger class‐based differences.

We conduct several robustness checks to further interrogate our results so far. First, we check if our main results are affected unduly by individual countries. We run jackknife analyses that systematically drop one country from the sample at a time and re‐estimate the models. Results from these analyses and jackknife standard errors yield findings that are substantively very similar to those discussed above. Additionally, we explore the impact of outlier countries by computing Cook's distance and dfbeta values for each country‐level inequality predictor. Excluding outlying countries does not change the substantive findings discussed above.

Second, we explore alternative measures of social class despite the strengths of a subjective measure of social class (Oesch and Vigna 2023). We use two alternative measures of class in robustness checks as discussed in the data section above: one is occupation‐based and the other is income‐based. As discussed above, the sample sizes in these analyses are considerably smaller due to missing data/questions across WVS waves.

**FIGURE A1:**
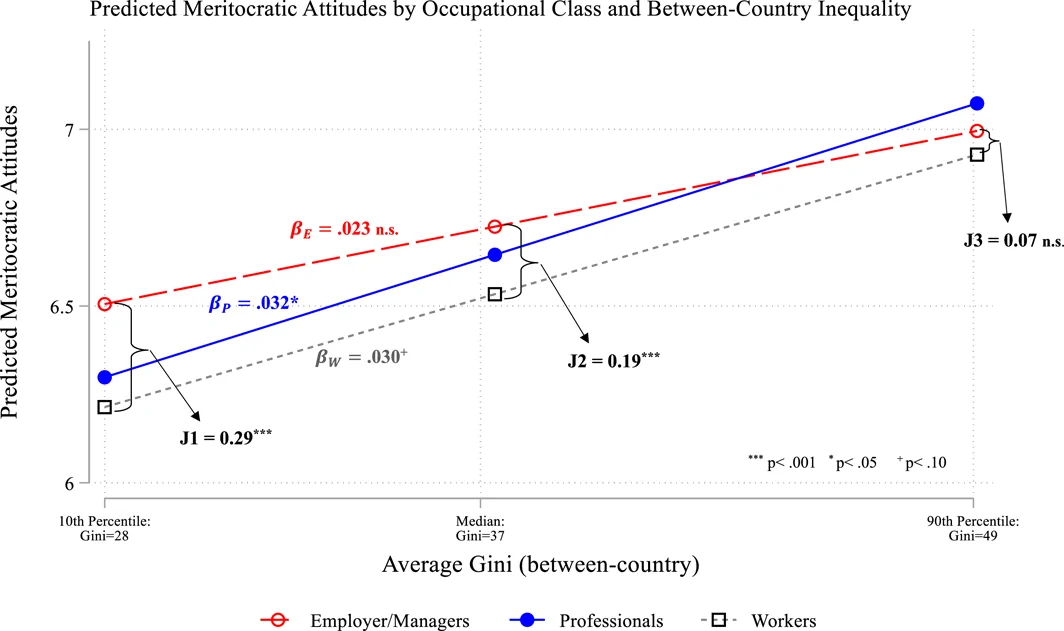
Predicted meritocratic attitudes by occupational class and between‐country inequality.

**FIGURE A2:**
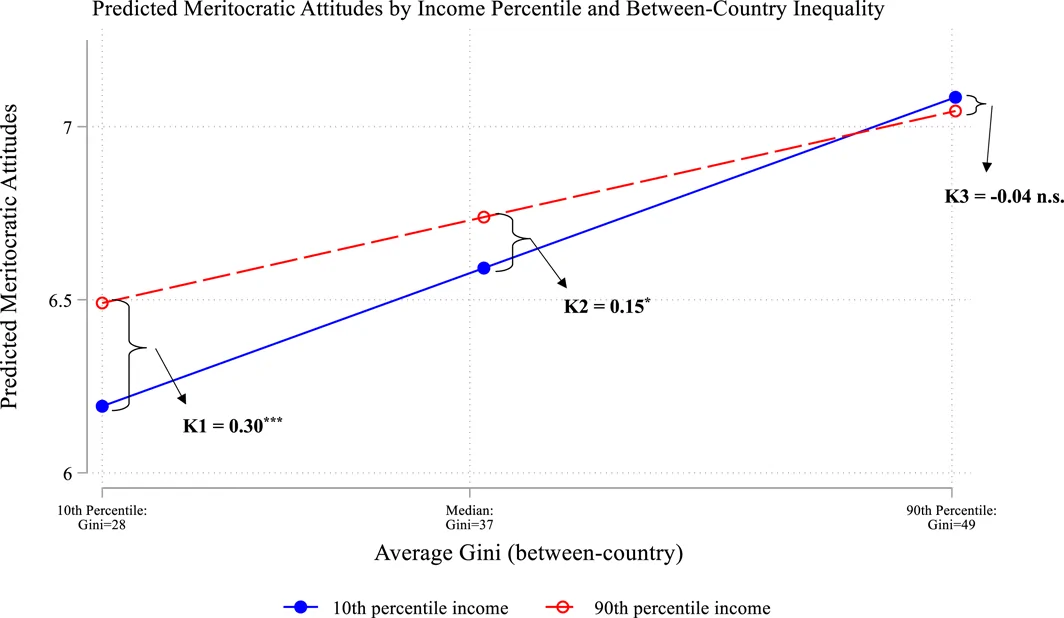
Predicted meritocratic attitudes by occupational class and between‐country inequality.

Table 2 displays the results from analyses that use these other measures of class. The findings are broadly quite similar to those from Table 1. According to Model 4, relative to employers and managers, both professionals and workers hold less meritocratic views. This is consistent with classic interest‐based explanations. Furthermore, we find evidence that these class‐based differences are smaller in more unequal contexts (i.e., higher between‐country inequality). Figure A1 in the appendix graphically illustrates these patterns. Just like the analyses with subjective class identification, these findings lend support to relative power theories of meritocratic views. Also similar to the main analyses, we find that declines in inequality are associated with an *increase* in meritocratic attitudes while increases in inequality are not related to changes in meritocratic attitudes. There do not seem to be substantial class differences in these longitudinal asymmetrical relationships except for some evidence that professionals respond more strongly to declines in inequality than do employers and workers. When considering the income‐based measure of class in Model 5, the results are again similar to the main results. Individuals at higher income percentiles tend to harbor more meritocratic views and these class‐based differences are smaller in more unequal countries (between‐country inequality) (see Figure A2 of appendix). Additionally, inequality declines are associated with increased meritocratic views but inequality increases do not seem to be consistently related to meritocratic views. Like the main results, there is some evidence that the positive relationship between inequality‐increase and meritocratic attitudes is stronger for higher income individuals.

**TABLE 2:** Coefficients from mixed effects regression of meritocratic attitudes with other class predictors.

	(4)		(5)	
	*β*	(SE)	*β*	(SE)
Country‐level predictors				
GDP per capita (ln) (between‐country)	−0.173	(0.168)	−0.148	(0.167)
GDP per capita (ln) (within‐country)	−0.738	(0.451)	−0.938	(0.559)
Gini average (between‐country)	0.020	(0.016)	0.028^+^	(0.016)
Gini decrease (within‐country)	0.315^***^	(0.087)	0.283^**^	(0.103)
Gini increase (within‐country)	0.048	(0.062)	0.069	(0.085)
Occupational class (ref. Employers/managers)				
Professionals^a^	−0.084*	(0.035)		
Workers^a^	−0.194***	(0.032)		
Gini average (between) × Occupational class				
× professionals	0.012**	(0.004)		
× workers	0.010**	(0.004)		
Gini decrease (within) × Occupational class				
× professionals	0.118*** ^,^ **	(0.031)		
× workers	0.026	(0.030)		
Gini increase (within) X Occupational class				
× professionals	0.038	(0.027)		
× workers	0.036	(0.024)		
Income percentile (from donnelly and pop‐eleches)			0.176^*^	(0.071)
Gini average (between) × income percentile			−0.021^*^	(0.008)
Gini decrease (within) × income percentile			0.002	(0.064)
Gini increase (within) × income percentile			0.086^+^	(0.046)

Full set of controls (see note)	Y		Y	
Observations	95,971		107,177	
Country‐waves	89		87	
Countries	65		63	

*Note:* Standard errors in parentheses. Models include individual controls for age, gender, education, religious person, employment status, and parental status in addition to region and year fixed effects. The model also includes random intercepts for country and country‐years as well as random slopes for individual class predictors involved in cross‐level interactions.

^a^ Main effects of interacted variables are computed with other relevant continuous predictors held at sample means.

^+^ *p* < 0.10.

^*^ *p* < 0.05.

^**^ *p* < 0.01.

^***^ *p* < 0.001 (two‐tailed test).

To sum up, we find that lower class individuals express less support for meritocratic narratives compared to upper‐class individuals, and these class‐based cleavages in meritocratic attitudes are smaller in countries that are more unequal on average. Lower class individuals in particular express markedly more meritocratic views in more unequal countries compared to those in egalitarian countries. When looking at the within‐country asymmetrical temporal relationships, our results suggest that falling inequality is associated with increased meritocratic views across class lines with no changes to class‐based differences. However, rising inequality is not as strongly related to changes in these views. In fact, we find some limited evidence that rising inequality is associated with increased meritocratic attitudes among higher‐class individuals only, which expands class‐based differences in merit attitudes.

## 7 Discussion and Conclusion

This study builds on past literature and presents findings that provide a more nuanced picture of meritocratic attitudes compared to previous studies. We first reproduce the familiar finding that among individuals who identify as low‐ or working‐class, meritocratic attitudes tend to be stronger in countries that are on average more unequal, so that class‐based differences are also smaller in these contexts. Similar to recent studies (Solt et al. 2016; Morris et al. 2022), this finding lends support to theories of relative power which suggest that in more unequal contexts, the rich have greater resources and abilities to disseminate meritocratic narratives and the poor are less able to produce counter‐narratives. Additionally, this finding is also consistent with theories that emphasize the role of segregated social and physical spaces in undermining people's ability to accurately perceive the extent of economic inequality in highly unequal societies (Massey and Tannen 2016; Mijs 2021; Mijs and Adaner 2024).

However, analyzing the *asymmetrical temporal* dimensions of the relationship between inequality and meritocracy yields different results. We find that *declining* inequality is associated with increases in meritocratic attitudes across class lines. This pattern is partly consistent with interest‐based accounts of activated class conflict which suggest that falling inequality might enhance the legitimacy of the economic system, especially for lower class individuals who accurately perceive the decline in inequality (Newman et al. 2015). However, our results do not suggest that class‐based differences in attitudes change significantly with declining inequality.

In contrast, we find no evidence that *rising* inequality is associated with declining meritocratic attitudes among lower class individuals. Instead, we uncover no link between rising inequality and merit attitudes among these groups. This is inconsistent with activated class conflict theory which suggests that rising inequality should undermine meritocratic views among lower classes and also inconsistent with recent studies which find that rising inequality increases concern about inequality among residents (Wiesner 2025; cf. Waight and Goldstein 2025). However, we do find some limited evidence that rising inequality is linked to *increased* meritocratic attitudes among upper‐class individuals, resulting in widening class‐based differences in these attitudes. This finding is consistent with the notion that upper‐class individuals might embrace meritocratic ideas as their class consciousness heightens with rising inequality.

In sum, our analyses yield novel findings that demonstrate (1) the utility of disentangling the various components of the inequality‐meritocracy relationship, and (2) how distinct and competing theories can explain different components of this relationship (i.e., cross‐sectional vs. asymmetrical temporal components). It is worth emphasizing the insights gained by relaxing the assumption of temporal symmetry the relationship by contrasting our findings with recent research. Bartram (2023) documents a negative temporal relationship between income inequality and meritocratic attitudes and interprets this as suggesting that rising inequality is associated with declining merit attitudes. However, our results suggest that the negative temporal relationship possibly only holds for *declining* inequality, not increasing inequality. Similarly, Wiesner (2025) detects a positive temporal relationship between income inequality and people's perceptions of and concern about inequality but the findings here point to the possibility that this relationship might not be symmetrical. We also note that our findings related to asymmetry have parallels to Mijs et al. (2022) which uses a Dutch sample of respondents to examine the relationship between subjective social mobility and meritocracy. They find that while experiencing upward mobility is associated with stronger meritocratic beliefs, *downward* mobility is not associated with weakened meritocratic beliefs. Again, this asymmetrical relationship operates in ways that seem to reinforce meritocratic views. It is also important to note that while Liu and Wang (2026) document contrasting relationships between education and meritocratic attitudes depending on the level of contextual inequality, our results suggest a somewhat more consistent relationship between class and meritocracy across the inequality spectrum: lower‐class individuals are less meritocratic in their outlook in all but the most unequal countries, where these class‐based differences fade. Finally, our findings are consistent with past research that emphasizes the importance of people perceiving inequality as unfair to precipitate concern for inequality and support for redistribution (Ahrens 2022). The mere existence of or increase in inequality do not seem to provoke skepticism of meritocratic narratives.

What are the wider implications of the findings documented here? For policymakers and activists concerned with curtailing inequality, the results suggest an uphill task at hand. One might expect low‐ and working‐class groups to be a “natural” constituency for policies designed to reduce inequality. Political organizations and social movements have historically used class‐based politics to organize this constituency into a political force that delivers redistributive policies (Calnitsky and Ella Wind 2024). However, class seems to matter *less* in shaping meritocratic attitudes in precisely those high‐inequality contexts where lower class groups have the most to gain from the politics of redistribution. Further, while falling inequality is readily met with rising meritocratic attitudes among all classes, the inverse does not seem to be true. Put differently, temporal changes in inequality have an asymmetric relationship with people's attitudes in ways that further entrench meritocratic narratives across class lines. Consequently, we suggest that a class‐based politics of redistribution that takes for granted the support of lower‐class groups seems unlikely to succeed. Rather, the findings here indicate that political organizations and movements interested in inequality alleviation have the more difficult task of countering dominant narratives and cultivating a class consciousness that links the material circumstances of lower‐class groups to structural narratives about inequality.

## Funding

The authors have nothing to report.

## Conflicts of Interest

The authors declare no conflicts of interest.

## Data Availability

The data underlying the analyses in this study are publicly available from the World Values Survey website (https://www.worldvaluessurvey.org/wvs.jsp) (Inglehart et al. 2020), the Standardized World Income Inequality Database (SWIID) (https://fsolt.org/swiid/) (Solt et al. 2016), and the World Bank (https://data.worldbank.org/) (World Bank 2024).

## Endnotes

^1^
For example, Waight and Goldstein (2025) show that fewer Americans perceived inequality to be growing in the 1990s and 2000s precisely during an upswing in inequality.

^2^
The samples in models with the occupation‐ and income‐based measures of class do not contain data from waves 6 and 7. This reduces the sample to about 87–89 country‐waves and 63–65 countries.

^3^
Additionally, Donnelly and Pop‐Eleches (2018) state that a number of WVS questionnaires for various country‐years do not associate the deciles of this income measure to country‐specific income brackets, which means that respondents have to rely on their own perceptions of where they fall in the income distribution.

^4^
For *t = 1*, both $x_{jt}^{+}$ and $x_{jt}^{-}$ are set to 0 consistent with Allison (2019).

^5^
We follow Allison (2019) in mean‐deviating these $z_{jt}^{+/-}$ variables in order to ensure that we are only relying on temporal changes in the Gini during the sample time period being studied rather than incorporating time‐invariant country‐specific historical changes in the Gini that predate the sample period. See p.8 of Allison (2019) for more information.

^6^
The “West” includes USA, Canada, Western Europe, Australia, and New Zealand.

^7^
We take the average of the last 3 years to ensure that the results are not unduly affected by the “noise” associated with yearly fluctuations. However, this is likely a minor concern given that for both the Gini and GDP per capita, the 3‐year lag averages are almost perfectly correlated with the single year lag values (*r ≥* 0.97 for Gini and *r ≥* 0.99 for GDP per capita).

## Appendix

**TABLE A1:** Descriptive statistics.

	Mean	SD	Min	Max
Outcome				
Belief in meritocracy	6.74	2.85	1	10
Focal Predictors				
Identify as lower or working class?	0.41		0	1
Gini coefficient (country‐level)	37.86	8.67	22.01	63.49
Controls				
GDP per capita (ln) (country‐level)	9.55	0.96	6.74	11.49
Age	42.31	16.38	15	103
Female	0.52		0	1
Religious?	0.65		0	1
Employed?	0.56		0	1
Married?	0.65		0	1
Parent?	0.72		0	1
Primary education or less	0.26		0	1
Secondary education	0.48		0	1
Tertiary education	0.26		0	1

*Note: N* = 249,251; 184 country‐years, 83 countries.

**TABLE A2:** List of countries and waves included in analyses.

	WVS waves					WVS waves			
	3	5	6	7		3	5	6	7
Albania	X				Lebanon			X	
Algeria			X		Lithuania	X			
Argentina	X	X		X	Malaysia		X	X	X
Armenia	X		X	X	Mali		X		
Australia	X	X	X	X	Mexico	X	X	X	X
Azerbaijan	X		X		Moldova	X	X		
Bangladesh	X			X	Mongolia				X
Belarus	X		X		Morocco		X	X	
Bolivia				X	Myanmar				X
Brazil	X	X	X	X	Netherlands		X	X	X
Bulgaria	X	X			New Zealand	X	X	X	X
Burkina Faso		X			Nigeria	X		X	X
Canada		X		X	Norway	X	X		
Chile	X	X	X	X	Pakistan			X	X
China	X	X	X	X	Palestine			X	
Colombia			X	X	Peru	X	X	X	X
Cyprus		X	X	X	Philippines	X		X	X
Czech Republic	X			X	Poland		X	X	
Dominican Republic	X				Puerto Rico	X			X
Ecuador			X	X	Qatar			X	
Egypt		X	X	X	Romania	X	X	X	X
El Salvador	X				Russian Federation	X	X	X	X
Estonia	X		X		Rwanda		X	X	
Ethiopia		X			Serbia		X		X
Finland	X	X			Singapore			X	X
France		X			Slovak Republic	X			X
Georgia		X	X		Slovenia		X	X	
Germany	X	X	X	X	South Africa	X	X	X	
Ghana		X	X		Spain	X	X	X	
Greece				X	Sweden	X	X	X	
Hong Kong				X	Switzerland	X	X		
Hungary	X	X			Thailand		X	X	X
India	X	X	X		Trinidad & Tobago		X		
Indonesia		X		X	Tunisia			X	
Iran		X		X	Turkey	X	X	X	X
Iraq			X		Ukraine	X	X	X	X
Italy		X			United Kingdom		X		
Japan		X	X	X	United States	X		X	X
Jordan			X	X	Uruguay	X	X	X	X
Kazakhstan			X	X	Vietnam		X		X
South Korea	X	X	X	X	Zambia		X		
Kyrgyz Republic			X	X	Zimbabwe			X	X
Latvia	X								

*Note:* Wave 3: 1994–98, Wave 5: 2005–09, Wave 6: 2010–14, Wave7: 2017–22.
